# A multisensory perspective of working memory

**DOI:** 10.3389/fnhum.2015.00197

**Published:** 2015-04-21

**Authors:** Michel Quak, Raquel Elea London, Durk Talsma

**Affiliations:** Department of Experimental Psychology, Ghent UniversityGhent, Belgium

**Keywords:** working memory, multisensory processing, multisensory integration, attention, top-down, bottom-up

## Abstract

Although our sensory experience is mostly multisensory in nature, research on working memory representations has focused mainly on examining the senses in isolation. Results from the multisensory processing literature make it clear that the senses interact on a more intimate manner than previously assumed. These interactions raise questions regarding the manner in which multisensory information is maintained in working memory. We discuss the current status of research on multisensory processing and the implications of these findings on our theoretical understanding of working memory. To do so, we focus on reviewing working memory research conducted from a multisensory perspective, and discuss the relation between working memory, attention, and multisensory processing in the context of the predictive coding framework. We argue that a multisensory approach to the study of working memory is indispensable to achieve a realistic understanding of how working memory processes maintain and manipulate information.

## A Multisensory Perspective of Working Memory

In everyday life we experience a continuous stream of information that we perceive through sight, sound, smell, taste, and touch. Even though this experience is mostly multisensory, that is, we receive information from multiple senses simultaneously, psychological research has primarily focused on studying our senses in isolation. While we are beginning to understand how our senses interact at various stages of processing (for an overview see, e.g., Wallace et al., 1993; Beauchamp, 2005; Ghazanfar and Schroeder, 2006; Stein and Stanford, 2008; Klemen and Chambers, 2012) it is still heavily debated whether the higher-order mental representations that are derived from these sensory inputs still contain modality- specific information or not. For instance, in working memory, research has focused on resolving whether information is memorized in the form of separate, modality or domain specific representations (Baddeley and Hitch, 1974; Schneider and Detweiler, 1988), or as integrated representations (Atkinson and Shiffrin, 1968; Cowan, 2001).

Multisensory processing refers to the interaction of signals arriving nearly simultaneously from different sensory modalities. This implies that information from one modality can influence information processing in another modality. Information from different sensory modalities can also be combined into a single multisensory event, a process that is referred to as multisensory integration (Stein et al., 2010). In accordance with the suggestions of Stein et al. (2010) we will use the terms “modality-specific” or “cross-modal” when describing the properties of objects and “unisensory” or “multisensory” when referring to neural or behavioral processes associated with a single or multiple sensory modalities.

The aim of this paper is to discuss the current status of research on multisensory processing and the implications of these findings for our theoretical understanding of working memory. To do so, we will focus on reviewing working memory research conducted from a multisensory perspective. We will argue that a multisensory approach to the study of working memory is indispensable to achieve a realistic understanding of how working memory processes maintain and manipulate information.

## Working Memory and the Multisensory Brain

In their seminal work, Atkinson and Shiffrin (1968) devised a model for the flow of information in human memory, which subsequently became known as the modal model. They suggested that environmental information is processed by various modality-specific sensory registers before it is combined into a single, modality-independent, or more formally *amodal*, percept and transferred into a short-term store. According to this view, the short-term store is an amodal, general-purpose mechanism. Atkinson and Shiffrin referred to this mechanism as “working memory”, as it was considered to be responsible for a variety of operations, such as the selection, manipulation, and rehearsal of the memorized items.

A few years later, Baddeley and Hitch (1974) proposed a multiple-component model of working memory where information is assumed to be stored in two domain-specific subsystems (the phonological loop and the visuo-spatial sketchpad) that are directed by a general control mechanism (the central executive). The phonological loop is responsible for short-term maintenance of speech-based and acoustic items. The visuo-spatial sketchpad maintains visually and/or spatially encoded items. In contrast to Atkinson and Shiffrin’s (1968) idea of a domain-independent (i.e., amodal) store, Baddeley and Hitch (1974) assume that information (e.g., verbal or spatial) is maintained in its corresponding domain-specific store.

Over the years it has become clear that information from different domains showed more interaction in working memory than one would expect from a strongly domain-specific perspective (e.g., Jiang et al., 2000; Logie et al., 2000; Prabhakaran et al., 2000). An episodic buffer was added to Baddeley and Hitch (1974) original working memory model to account for, amongst other things, the apparent interaction between phonological and visual processes (Baddeley, 2000). The episodic buffer can be conceived as an amodal storage component, which was estimated to hold up to four chunks of information. Additionally, it was proposed to act as a link between all the other working memory components described above. For this revised model, Baddeley (2000) suggested that the episodic buffer integrates memory traces that may originate from different senses into a coherent perceptual scene.

On the basis of several studies, Postle (2006) has proposed that the brain areas involved in sensory perception are also responsible for the short-term storage of sensory information. For instance, functional magnetic resonance imaging (fMRI) studies showed object-specific memorization effects for faces in the posterior fusiform gyrus (e.g., Druzgal and D’Esposito, 2003; Ranganath et al., 2004), an area considered to be vital for face recognition. Postle and D’Esposito (1999) found activity related to memorization of visual object location and depiction in ventral temporal and occipital visual brain areas. Similarly, event-related potential (ERP) modulations can be seen in posterior and occipital recording sites during short-term memorization of visual objects contralateral to the to-be-remembered objects (e.g., Klaver et al., 1999; Vogel and Machizawa, 2004). Such findings (for an overview see, Postle, 2006; D’Esposito and Postle, 2015) indicate that memorizing modality-specific sensory information involves the same brain areas as those involved in the initial sensory processing of that information. This idea is compatible with the classical view that integration of the senses would take place at a later stage of processing, after initial unisensory processing has taken place (see Talsma, 2015, for a discussion). Indeed, using neurophysiological methods with animals (e.g., Wallace et al., 1993; Fuster et al., 2000) and fMRI with humans (e.g., Calvert et al., 2000; Wright et al., 2003; Beauchamp et al., 2004) several higher-order brain areas have been identified that seem to be dedicated to integrating information from multiple unisensory sources. Brain areas typically regarded as multisensory in the human brain can for example be found in the lateral occipital-temporal cortex, such as the superior temporal sulcus (STS; Beauchamp, 2005).

An increasing number of studies now suggest, however, that multisensory processing can already take place in brain areas that were considered to be strictly unisensory (see for a review, Foxe and Schroeder, 2005; Macaluso and Driver, 2005). For example, Giard and Peronnet (1999) found multisensory ERP effects as early as 40 ms post-stimulus over occipital scalp areas, suggesting that multisensory interactions take place much earlier than previously assumed. Using fMRI, Foxe et al. (2002) showed integration related effects of auditory and somatosensory stimuli within a region of the auditory cortex previously thought to be unisensory. This brain area was more strongly activated by multisensory stimuli than what might be expected on the basis of a mere summation of either auditory or tactile stimulation alone. Likewise, Dionne et al. (2010) found increased BOLD signal in the right primary somatosensory cortex during a delayed sensory-to-motor task for cross-modal visual-somatosensory stimuli compared to modality-specific stimuli.

These findings also have implications for the memorization of multisensory information. If indeed, as Postle (2006) proposes, the brain areas responsible for perceptual processing are the same as those involved in memorization, and if multisensory effects can already be observed in the primary sensory cortices, then we would expect that cross-modal information is stored as a unified representation in working memory. We specifically aim to focus on the questions regarding how multisensory information is encoded in working memory and whether we memorize the individual unisensory representations separately and integrate them at a later stage, or whether they are memorized as part of an integrated, multimodal representation instead.

## Feature Binding in Working Memory

To fully understand the importance of considering working memory from a multisensory perspective, it is necessary to discuss how information is organized within working memory. An important question here is whether each feature of an object is remembered separately or not (e.g., Luck and Vogel, 1997; Klaver et al., 1999; Vogel et al., 2001, 2005; Wheeler and Treisman, 2002; Olsson and Poom, 2005; Luria et al., 2010; Diamantopoulou et al., 2011; Luria and Vogel, 2011). For example, Luck and Vogel (1997) used a change detection task to examine the capacity of working memory for visual objects. Participants were presented with an array of stimuli, which they had to remember during an interval without the stimuli being present. After this retention interval a second array was presented and participants responded by indicating whether any visual changes had occurred between the second and the first array. Varying the number of visual objects that need to be memorized allows estimating the capacity of visual working memory. Luck and Vogel (1997) found that capacity was limited to approximately four objects, regardless of the number of feature dimensions, or individual features that needed to be remembered per object. This led them to conclude that visual working memory has an object-based and not a feature-based organization. It is important to note that these findings have not been replicated (Oberauer and Eichenberger, 2013; Hardman and Cowan, 2015). At the very least this suggests that feature binding can, but does not always, occur automatically.

Interestingly, research has shown that an asymmetry exists in binding the visual and spatial features of an object. Multiple studies have shown that processing the visual features of an object automatically bind this object to its spatial location (e.g., Jiang et al., 2000; Olson and Marshuetz, 2005). However, processing an object’s spatial location does not result in the automatic binding of that object’s visual features (Jiang et al., 2000). While these findings show that binding of multiple features can occur within the visuo-spatial domain, other studies have shown that binding of features can even occur across domains.

Prabhakaran et al. (2000) showed that participants memorized verbal and spatial information in an integrated fashion. Participants in this study performed faster and more accurate on a verbal-spatial delayed-match-to-sample task when the probe was a letter-location combination that was presented together in the sample array compared to a letter-location combination that was presented separately. The findings on binding of verbal and spatial information have been replicated and extended in multiple studies (Bao et al., 2007; Campo et al., 2008, 2010; Elsley and Parmentier, 2009; Guérard et al., 2013; Meier et al., 2014). For example, Bao et al. (2007) found that switching attention between verbal and spatial features was faster when they were features from one object than when they were features from separate objects. Additionally, Guérard et al. (2013) showed that phonological similarity of verbal material can carry over to the recall of spatial locations in a combined verbal-spatial serial recall task. Participants were sequentially presented with letters in specific locations and were asked to either recall the order of spatial locations shown or the order of letters shown. They found that the harmful effect of phonological similarity on verbal recall carried over to spatial recall, but that the harmful effect of spatial complexity on spatial recall did not carry over to verbal recall. While the question remains under which exact circumstances automatic binding or integration of cross-domain information occurs, the asymmetry found in visual feature and location binding as well as verbal and spatial binding, suggest that the automatic integration of information across domains can occur.

## Multisensory Working Memory Representations

Despite the evidence for integration of information from different domains, surprisingly little research has examined how multisensory information is represented in working memory. One of the first studies to use cross-modal stimuli was done by Thompson and Paivio (1994). Participants memorized three different types of items: visual, auditory, or audiovisual for a later free-recall test. Thompson and Paivio found an improvement of free recall of cross-modal audiovisual stimuli compared to modality-specific, audio or visual stimuli. This superior audiovisual performance was not simply due to the double presentation of information in audiovisual conditions (audio and visual dual presentation), because picture-picture and sound-sound dual presentation conditions did not yield a similar improvement. When pictures in the picture-picture dual presentation condition were two different exemplars of the same item a slight improvement in free recall was found but audiovisual performance still resulted in higher recall rates. Goolkasian and Foos (2005) also found that recall rates were higher for picture/spoken word and written/spoken word dual presentation conditions compared to the double visual presentation of pictures and written words. These findings suggest that the improved memory performance is due to the combination of information from different modalities and not because of the redundancy of the information itself.

In the multisensory literature, additive effects, such as for example linear increases of brain activity for multisensory stimuli (For an overview see; Calvert, 2001), are considered to be exemplary of multisensory processing. By contrast, in working memory research, similar additive effects, such as an increase in capacity for audiovisual material compared to modality-specific material, are considered evidence for the independence of the two modalities. For example, the advantage of cross-modal object recall, in the study of Thompson and Paivio (1994) was explained by Paivio (1971, 1986) “dual coding” theory. This theory states that a memory trace for a cross-modal stimulus is a combination of the independent sensory traces that were encoded, which in turn can be recalled separately when the task so requires. While information from different modalities can interact to provide certain behavioral benefits, this information is in fact independent.

Originally, the dual coding theory was developed to explain the independent, simultaneous processing of verbal and non-verbal information, but has later also been used to explain the independent, simultaneous processing of auditory and visual information. It is important to note that these forms of information can interact. Verbal information can be both visual (e.g., written words) and/or auditory (e.g., spoken words), and nonverbal information can also be visual (e.g., complex visual scenes) and/or auditory (e.g., white noise). We can make a distinction between the format of a working memory representation, i.e., the sensory modality in which the information is perceived and/or processed (e.g., auditory—visual), and the content of the representation, i.e., the actual information that is transferred (verbal–non-verbal). For example, when memorizing an array of blue squares or a picture of a cat, it might be more efficient to memorize this verbally as the verbal code “blue squares” or “red cat”. However, when the task requires one to describe the exact spatial location of each square, or point out a specific cat in an array of red cat pictures, it would be more efficient to use a visual code. We assume that information is processed in the format code that is most optimal for the current task. This implies that multiple format codes might be used for one and the same object, if that is more effective for memorizing that object.

Delogu et al. (2009) investigated how verbal and non-verbal auditory, visual, and audiovisual material is encoded in working memory. Participants were tested on immediate serial recall for sequentially presented visual, auditory, or audiovisual stimuli in either a non-verbal or verbal condition. In the non-verbal condition, stimuli were either pictures, environmental sounds, or a combination of both, and in the verbal condition, stimuli were either written words, spoken words, or a combination of both. Results showed that in the non-verbal condition serial recall for audiovisual stimuli was higher than recall for auditory or visual stimuli. In the verbal condition, recall for audiovisual material was still higher than recall for visual material, but auditory and audiovisual recall did not differ. The authors also found that preventing participants from articulating reduced memory performance in both the verbal and non-verbal conditions. This suggests that both in the verbal and in the non-verbal presentation conditions, the actual content of the representation was encoded in a verbal code. Furthermore, the verbal content seemed to play a key part in memorizing the stimuli in all conditions. This shows that the format in which information is presented is not necessarily the format in which the information is encoded. For example, when a participant is presented with an auditory stimulus of a meowing cat, it is possible that this sound calls forth a picture of a cat, or the word “cat”, which is then kept in working memory instead of the auditory features of the original meowing sound that was presented. It is a requirement that the participant recognizes the presented sound as the meowing produced by a cat in order to “recode” the sound into a visual or verbal representation. This requires semantic information from long term memory to be integrated with the working memory representation. Delogu et al. (2009) concluded that their findings are compatible with Baddeley’s (2000) working memory model where the existence of an episodic buffer integrates information from different modalities and combines this with semantic information from long term memory. Other studies have also shown the influence of semantic information from long term memory on visual working memory object representations (e.g., Olsson and Poom, 2005; Diamantopoulou et al., 2011) suggesting that information outside the pure visual domain can affect early visual object working memory. Similarly, Darling et al. (2012) found that accuracy on a digit serial recall task improved when the locations of presented digits matched the spatial configuration of a typical, numeral keypad (as found on a telephone or television remote) in a process they call visuospatial bootstrapping. They confirmed that this effect was due to the integration of the typical keypad representation from long-term memory with the working memory representation and not only to the binding between verbal and spatial information.

Thus far, the main goal of the studies discussed above was to provide insights into the dual code theory (Paivio, 1971, 1986) and/or the multiple component theory (Baddeley and Hitch, 1974; Baddeley, 2000) mainly by looking at recall performance for a wide variety of stimuli. To better understand how multisensory information interacts in working memory we can look at working memory capacity for cross-modal objects. As mentioned before, estimates of working memory capacity for features and objects have been used to infer that visual working memory representations are object based (Luck and Vogel, 1997). Likewise, by assuming that not only features within a modality but also across modalities are integrated into object representations, examining the number of cross-modal objects one can hold in memory compared to modality-specific objects could give insight into the organization of multisensory working memory. For instance, Saults and Cowan (2007) found that working-memory capacity for audiovisual material can exceed working-memory capacity for modality-specific material under certain conditions. In a series of five experiments, participants were presented with visual arrays of four to eight colored squares and auditory arrays of four spoken digits. They were instructed to memorize the visual array, the auditory array, or both. Interestingly, the performance advantage for audiovisual arrays disappeared when masks were used to block access to previously formed sensory memory traces. In this case, capacity for cross-modal stimuli was as high as the capacity of the highest modality-specific object, indicating that memory traces from an accessory sensory memory (echoic and/or iconic memory) contributed to the improvement of task performance. Since auditory and visual information did not additively contribute to memory performance when sensory memory traces were excluded, Saults and Cowan (2007) concluded that auditory and visual information share a common storage. Fougnie and Marois (2011) contested this interpretation by arguing that the formula used by Saults and Cowan (2007) to estimate the maximum number of object representations one can hold in working memory, might not adequately reflect the combined capacity of modality-specific stores. Fougnie and Marois argued that one item of auditory information generally places a larger load on memory than one item of visual information, suggesting that these modality-specific differences should be weighted accordingly in such a capacity estimate. Using an adapted formula in a series of three experiments, they found that even when using masks to exclude contributions of sensory memory traces, capacity for cross-modal items was superior to the capacity for modality-specific items. Contrary to Saults and Cowan (2007), they concluded that auditory and visual objects were stored in their own respective stores and contributed to performance without interfering.

Overall, there seems to be a performance benefit for the memorization of audiovisual stimuli compared to the memorization of modality-specific stimuli. It remains under debate, however, whether this benefit exists because these stimuli are integrated into a new amodal representation or because the independent storage of auditory and visual information contributes to performance in an additive fashion because they do not interfere. At this time the same effect is used to argue for both sides of the debate. Where some see the additive performance of audiovisual objects as proof for an interaction or even integration of information in working memory (e.g., Delogu et al., 2009), others see it as proof that sensory information is memorized in its own separate store (e.g., Fougnie and Marois, 2011).

In addition to examining performance benefits for the combination of auditory and visual processing, we can also study the disruption of processing for the combination of auditory and visual information. In traditional working memory research, interference paradigms have been used to show a double dissociation between two separate processing mechanisms. Meaning that when two processes use the same underlying system, interference will occur which impairs performance on both processes. The disruption of performance between modalities is referred to as cross-modal interference and would suggest that information from the different modalities interact at a certain level. For multisensory working memory this could mean that information from different modalities is maintained in a single, multisensory store. Evidence for cross-modal interference is still somewhat ambiguous, however. For instance, using a visual-pattern-recall and auditory-digit-recall dual task, Cocchini et al. (2002) did not find evidence for cross-modal interference on performance accuracy in working memory. The absence of such interference suggests that working memory operates in a domain-specific manner and is in accordance with the notion of parallel processing without interaction of information from different modalities. In contrast, Goolkasian and Foos (2005) showed that spoken words could interfere with the recall of pictures and written words when using long sequences of incongruent dually presented items. Likewise, Morey and Cowan (2004, 2005), did find cross-modal interference on performance accuracy when memory load was sufficiently high. They examined digit span using a verbal-visual dual task and found that participants showed interference for visual memory recall but only when the verbal load was sufficiently high (a load of 7 digits instead of 2). The interference patterns observed in audio-visual dual tasks are as of yet inconclusive on whether visual and auditory information share a limited capacity storage. Although interference paradigms could give us an answer on the question of whether information from different modalities share a limited capacity storage or not, they cannot answer whether the information from different modalities is integrated in this single storage, or maintained as independent modality-specific traces.

Thus far, research on multisensory working memory has shown that recall is better for cross-modal objects compared to modality-specific objects (Thompson and Paivio, 1994; Goolkasian and Foos, 2005; Delogu et al., 2009), working memory capacity is higher for cross-modal objects under certain circumstances (Saults and Cowan, 2007; Fougnie and Marois, 2011), and visual and auditory information can interfere with each other (Morey and Cowan, 2004, 2005; Goolkasian and Foos, 2005) but not always (Cocchini et al., 2002). Although a performance benefit for cross-modal objects is seen as evidence for integration in multisensory research, in working memory research it has traditionally been seen as evidence that modality-specific information from cross-modal objects is stored in separate stores. While we cannot definitively conclude that cross-modal objects are stored as fully integrated objects in working memory, it is apparent that cross-modal information interacts in working memory beyond what would be expected from modality-specific stores. The question is: at what stage or stages in the processing stream do these interactions occur?

## Multisensory Processing, Selective Attention, and Working Memory

To answer this question we turn to research on multisensory processing and selective attention. The insights gained from this research could also inform questions about working memory for multisensory stimuli. In fact, more and more researchers have challenged the idea that working memory and attention are two separate systems (Cowan, 2001; Awh et al., 2006; Olivers, 2008; Oberauer and Hein, 2012; Kiyonaga and Egner, 2013; Klaver and Talsma, 2013). For example, Olivers (2008) reviews evidence for the notion that working memory and attention share the same capacity, content and control processes, suggesting they might be two aspects of the same process. Likewise, Kiyonaga and Egner (2013) discuss the literature that examined the effects of external attention on working memory representations, as well as, the effects of working memory representations on directing selective attention. These studies indicate that a competitive interaction between working memory and selective attention exists, implying that they share a limited resource. Kiyonaga and Egner (2013) state that attention and working memory should no longer be regarded as two separate concepts, but instead as one concept, where attention can be directed externally (selective attention) and/or internally (working memory). The idea of working memory as internal attention is in line with Cowan’s (2001) original idea of working memory where a capacity limited focus of attention can shift between different levels of processing.

Given the above mentioned observations that working memory and attention are presumably two different aspects of the same underlying process, and considering that several studies have shown close ties between attention and multisensory processing, it is necessary to understand the implications of these ties for working memory. Instances where multisensory events guide and focus attention (also referred to as bottom-up effects) suggest an early integration of multisensory information, while instances where attention is needed for multisensory integration (also referred to as top-down effects) are indicative of late integration processes. There is evidence for both types of interaction between multisensory integration and attention. Factors that determine the predominance of either early and/or late interactions between information from different modalities are for example, task-relevancy (e.g., Busse et al., 2005), learned associations (e.g., Molholm et al., 2007), and saliency (e.g., Van der Burg et al., 2008).

An example of top-down influence of attention on multisensory integration was given by Talsma and colleagues (e.g., Senkowski et al., 2005; Talsma and Woldorff, 2005; Talsma et al., 2007). Using a rapid succession of task-relevant and irrelevant stimuli, they found that attention could influence the integration of cross-modal stimuli. Similarly, Alsius and colleagues (Alsius et al., 2005, 2007) have shown that attending elsewhere diminishes participants susceptibility to the McGurk illusion (McGurk and MacDonald, 1976). Based on these findings it appears that attending to the relevant, to-be-integrated stimuli is necessary to build a robust, integrated representation (Talsma et al., 2010).

However, evidence for bottom-up modulation of attention by multisensory integration has made it clear that multisensory processing can already happen in very early stages of perception (Giard and Peronnet, 1999; Molholm et al., 2002; Van der Burg et al., 2011). For instance, Van der Burg et al. (2011) presented dynamic displays consisting of line elements that randomly changed orientation. When a target orientation change was synchronized with a short, spatially uninformative tone, visual search was strongly facilitated as compared to when the tone was absent. The interpretation given to these results was that the tone and the synchronized orientation change were bound together into one coherent event, thereby forming a cross-modal singleton that “popped out” between the non-synchronized visual distractors. EEG data showed that this multisensory benefit was apparent as early as 50 ms post-stimulus onset and that the strength of this effect predicted the magnitude of the behavioral benefit during visual search, due to the auditory signal.

The findings above imply that both top-down (task-relevance and learned associations) as well as bottom-up (salience) processes are involved in multisensory integration. To resolve this apparent contradiction between a bottom-up view of multisensory processing, where early multisensory effects seem to *drive* attention, and a top-down view of multi-sensory processing, where attention seems to be *required* to integrate cross-modal objects, Talsma et al. (2010) proposed a unified framework of attention and multisensory processing. According to this framework, early pre-attentive processes can bind multisensory inputs together, but only when competition among the individual inputs is low. Thus, the early latency processes serve to cross-feed low-level information between the individual sensory cortices involved in the integration processes. Early interactions might serve to realign auditory and visual input signals. Auditory information might give temporal information to visual cortex whereas visual information might provide spatial information to auditory processing.

This pre-attentive early integration would, according to Talsma et al. (2010), only be possible, however, if the stimuli presented in one modality do not need to compete for processing capacity with other stimuli in that same modality. If there is competition among multiple stimuli in one modality, top-down attentional control may be required to filter out any stimulus that is not task relevant, thereby prioritizing those stimuli that are task relevant. Consistent with this view, Van der Burg et al. (2012) found that the earlier mentioned automatic capture by a synchronized cross-modal event can be modulated by the size of the attentional window, meaning that when participants were less focused the effect of the cross-modal pop out was stronger than when participants were forced to focus on a small cue before the synchronized cross-modal event. In conclusion, stimulus-driven, bottom-up processes can automatically capture attention towards multisensory events. Top-down attention can in turn facilitate the integration of multisensory information which leads to a spread of attention across sensory modalities.

Based on the previously mentioned idea that external attention and internal attention (working memory) are two aspects of the same process, findings in attentional research could be applied to working memory. It has been shown that spatial attention can actively influence working memory representations by facilitating encoding (Uncapher et al., 2011) and improving the recall of memorized representations (Murray et al., 2013). These effects are found not only within a single modality, but also across modalities. For instance, an auditory cue can draw attention to a visual object and* vice versa* (Spence and Driver, 1997; Koelewijn et al., 2009). Similar effects for working memory have been found by Botta et al. (2011). They examined the effect of visual, auditory, and audiovisual cues on working memory for arrays of colored squares in a change detection task. The cross-modal and modality-specific cues could either capture attention towards the hemifield which contained the to-be-remembered objects, or towards the opposite hemifield which contained the to-be-ignored objects. They found that audiovisual cues had a larger influence on performance accuracy than modality-specific visual or auditory cues. Memory accuracy was increased when an audiovisual cue was presented on the same side as the target and it was decreased when the audiovisual cue was presented on the opposite side. Both the facilitation and impairment of memory performance was larger for audiovisual cues compared to visual cues. Although these data do not directly address the question of how a cross-modal object is represented in working memory as such, they do tell us that multisensory information has a bottom-up effect on the subsequent memorization of a unisensory object.

Investigation of top-down effects of working memory on attention has revealed that working memory content can affect the allocation of visual selective attention (Olivers et al., 2006). In a multisensory context, Murray et al. (2004) found that discrimination accuracy of visual objects, presented 20 s after initial presentation, improved when the initial presentation was a picture-sound combination compared to a unisensory picture. EEG data revealed that the neuronal response to a cross-modal stimulus happened as fast as 60–136 ms and predominantly influenced activation in the right lateral occipital complex. Where a semantically congruent picture-sound combination increased discrimination accuracy on a second presentation, a pure tone decreased discrimination accuracy on a second presentation (Lehmann and Murray, 2005; Thelen et al., 2012). Thelen et al. (2015) replicate these earlier findings, while also showing the same effects in the auditory modality. Single-trial multisensory memories affect later auditory recognition. If cross-modal objects were congruent (visual and auditory information match semantically) accuracy was higher compared to unisensory stimuli but became worse if objects were incongruent or meaningless. Unisensory percepts seem to trigger the multisensory representations associated with them, suggesting at least a partially integrated storage in memory. Yet, it seems a multisensory representation stored in memory is only beneficial for memory performance when sounds and pictures are semantically congruent. These studies show that an internal representation is formed in which both the visual and auditory information is encoded. Moreover, they also indicate that information presented in a task irrelevant modality interferes with the task relevant representation. But although this still does not address the question of whether unisensory information is still accessible it does show that the original unisensory representations are closely related. Similar to the findings in research on attention and multisensory integration, it seems that top-down and bottom-up processes play an important part in the integration of cross-modal information in working memory representations.

## Predictive Coding and Multisensory Working Memory

One influential framework that can explain the intricacies of top-down and bottom-up interactions in multisensory memory is that of predictive coding. The predictive coding framework states that the brain produces a Bayesian estimate of the environment (Friston, 2010). According to this view, stochastic models of the environment exist in the brain, which are continuously updated on the basis of processed sensory information. Higher-order brain areas thus provide the lower areas with predictions (or in Bayesian terms “priors”) that influence the processing of ongoing sensory input. A strong mismatch between the prediction and the actual sensory input will then result in a major update of the internal model. Thus when we are in a complex environment with many stimuli competing for processing capacity, incongruence between the top-down predictions of the environment and the present incoming environmental information can determine the priority with which incoming stimuli need to be processed and integrated. The processed information changes the predictions and *vice versa*. Bottom-up sensory processing and top-down predictions mutually define each other continuously. In this way, the predictive coding view can explain how top-down and bottom-up processes interact in multisensory integration.

Talsma (2015) recently argued that the dynamic model of our environment provided by the aforementioned stochastic representations is essential to understanding the interaction between basic (multi)sensory processing on the one hand, and memory and attention on the other. For instance, Vetter et al. (2014) showed that actual auditory stimulation as well as imagined sounds could activate the visual cortex. Based on the predictive coding framework, these authors argued that visual cortex activation came about because either direct sensory information or a stored memory representation thereof could update the internal representation of the sound and therefore indirectly influence processing in visual cortex accordingly. This suggests that attention, memory, and multisensory processing are intrinsically intertwined. Similarly, Berger and Ehrsson (2013, 2014) showed that imagined sounds can mimic the effects of actual sounds in a number of well-known multisensory illusions, such as the bounce-pass illusion (Sekuler et al., 1997), the McGurk effect (McGurk and MacDonald, 1976), or the ventriloquist illusion (Howard and Templeton, 1966), and show independent of each other that visual cortex can be activated both by multisensory stimulation and by memory. Based on these findings, Talsma (2015) argued that despite the fact that several studies showed that auditory and visual inputs can interact at very early processing stages, the actual integration of the sensory inputs into a coherent mental representation occurs at later, higher-order processing stages.

An important consequence of applying the predictive coding framework is that our internal representation is assumed not only to be built on the basis of direct sensory input, but that it is also updated (and made consistent with) information stored in memory. Thus, attention is assumed to play an essential role in regulating how our sensory input is combined with these pre-existing representations stored in long-term memory. This is largely consistent with Cowan’s (2001) idea of the focus of attention, which is a part of activated long-term memory, as well as with Baddeley’s (2000) episodic buffer, although, Baddeley recently argued that attention in the form of the central executive was not necessary for the integration of multiple sources of information in the episodic buffer (Baddeley et al., 2011).

A further consequence of applying the predictive coding framework is that the internal representation is by definition always multisensory. Moreover, the active representation integrates all possible sources of information, including semantic information from long-term memory. Thus, even when only a unisensory stimulus is presented, associated representations will be activated as well. These can include information from other modalities, prior experience with the stimulus, or learned associations. Because the formation of this internal mental representation is an active process that influences ongoing processes in the sensory cortices, this model can explain why memory traces in one modality can be strengthened or corrupted by traces in another one. Furthermore, because the active representation sends feedback information to the low-level processes in sensory cortices it can be assumed that the original unisensory memory traces are still present albeit in a relatively fragile state.

## Multisensory Working Memory Representations in Current Models

The active internal environmental model as proposed by the predictive coding framework would be akin to what we would describe as a multisensory working memory representation. This memory representation does not only consist of information coming from different modalities but also includes information from long-term memory such as semantic knowledge or learned associations. Taking the previously mentioned example of memorizing a cat picture the multisensory representation includes not only the visual features of the cat, but also long-term semantic knowledge of cats, autobiographical knowledge (previous personal experience with cats), and information from modalities not presented with the picture (the sound a cat makes or the knowledge that its fur is soft to the touch). We assume that working memory has an amodal central storage component. Whether this is the main component of working memory as suggested by Cowan (2001) or a part of a bigger system like the episodic buffer in (Baddeley’s, 2000; Baddeley et al., 2011) remains a point for further investigation.

The predictive coding framework would suggest that incoming sensory information is constantly used to update the internal environmental model, implying that incoming stimuli tend to integrate into a coherent multisensory representation. This framework can also explain why working memory is amodal in some cases and modality specific in others. For instance, Postle (2006) argued that working memory for modality-specific stimuli occurs in the sensory cortices. Recently, Yonelinas (2013) suggested that high-resolution bindings are stored in the hippocampus that can be used to support perception and working memory, specifically in memorizing (combinations of) complex features. In the latter case it is plausible that the multisensory representation will be activated, whereas in the former case it is not. Based on this, one important implication of the predictive coding approach is that differences in task and stimulus complexity can yield rather drastically different outcomes. With this in mind a recommendation for future research would be to consider effects of task and stimulus complexity on working memory activation.

Based on the above mentioned framework, we assume that sensory cortices can retain small amounts of modality-specific information (as suggested by Postle, 2006) and that this information supports a multisensory memory representation in higher order areas (e.g., the hippocampus; Yonelinas, 2013). Whether working memory for a specific task involves the higher-order areas or the sensory areas to retain information for limited time depends on the task and the information that needs to be memorized. For example, simple flashes and beeps could be retained in the sensory areas, whereas more complex information would also require the higher-order areas. In that sense the sensory cortices would retain information in a manner similar to separate slave systems (Baddeley and Hitch, 1974) or the recently suggested peripheral storage (Cowan et al., 2014).

## Summary and Conclusions

In this paper we have reviewed recent developments in multisensory working memory research. Research has shown that cross-modal information interacts in working memory beyond what would be expected from the traditional modality-specific stores. Recall is better for cross-modal objects compared to modality-specific objects (Thompson and Paivio, 1994; Goolkasian and Foos, 2005; Delogu et al., 2009), working memory capacity can be higher for cross-modal objects than for unimodal objects (Saults and Cowan, 2007; Fougnie and Marois, 2011), and visual and auditory memory can interfere with each other (Morey and Cowan, 2004, 2005; Goolkasian and Foos, 2005). Furthermore, multisensory information has an effect on the subsequent memorization of a unisensory object (Botta et al., 2011) and multisensory memory representations can influence subsequent unisensory stimulus discrimination (Murray et al., 2004; Lehmann and Murray, 2005; Thelen et al., 2012, 2015). Taken together, these studies show that sensory representations in multiple modalities interact more with each other than can be explained by classical modal models.

Paivio’s (1971, 1986) dual coding theory states that although cross-modal information can interact it is in fact independent, because modality-specific information can still be retrieved in isolation. However, studies done by Thelen and colleagues (Thelen et al., 2012, 2015) show that this retrieval of modality-specific information from a cross-modal representation is more difficult than assumed, because a task irrelevant modality interferes with the task relevant representation. Moreover, higher-order representations of the external world built from memorized information have been shown to influence visual processing. Complex representations seem to be formed in working memory, consisting of the integration of several independent representations that can be sensory, and short- or long-term memory activations. Depending on task requirements either just the simple modal representation or the complex high-resolution binding of several features at once will become active. Therefore, we conclude that working memory is in essence multisensory, and that this must be taken into account to achieve a realistic understanding of how working memory processes maintain and manipulate information.

## Conflict of Interest Statement

The authors declare that the research was conducted in the absence of any commercial or financial relationships that could be construed as a potential conflict of interest.
